# Molecular characterization and epidemiology of carbapenem non-susceptible Enterobacteriaceae isolated from the Eastern region of Heilongjiang Province, China

**DOI:** 10.1186/s12879-018-3294-3

**Published:** 2018-08-22

**Authors:** Xue Gong, Jisheng Zhang, Shanshan Su, Yanjun Fu, Mingjia Bao, Yong Wang, Xiaoli Zhang

**Affiliations:** 1 Department of Microbiology, the First Affiliated Hospital of Jiamusi University, Jiamusi, Heilongjiang China; 2 Center for Disease Control and Prevention, Jiamusi, Heilongjiang China

**Keywords:** Molecular characterization, Epidemiologic typing, Carbapenem non-susceptible Enterobacteriaceae

## Abstract

**Background:**

The aim of this study was to elucidate the molecular epidemiology of carbapenem non-susceptible Enterobacteriaceae(CNSE) isolated in the Eastern region of Heilongjiang Province, China, and the mechanism of carbapenem resistance.

**Methods:**

A total of 53 CNSE isolates were collected in a grade-3 hospital in Heilongjiang province. Sensitivity to antibiotics was determined using the VITEK-2 Compact automatic system. The modified Hodge test (MHT) and modified carbapenem inactivation test (mCIM) were performed for phenotypic identification. Beta-lactamases gene were detected by Polymerase chain reaction(PCR) and DNA sequencing. The transfer of *bla*_NDM_ and *bla*_KPC_ was investigated through conjugation experiment. The clinical data of patients were retrospectively reviewed. Homology of Carbapenem-resistant *Klebsiella pneumoniae*(CRKP) was conducted by multilocus sequence typing (MLST).

**Results:**

CNSE were highly resistant to the majority of antimicrobial agents. The resistance rate was 100% for first, third, fourth generation cephalosporins and enzyme inhibitor compounds. Gentamicin and tobramycin recorded a resistance rate higher than 80%. Less than 30% resistance was detected for amikacin and levofloxacin. Among CNSE 52(98.1%) and 48(90.6%) of CNSE were positive for mCIM and MHT respectively. There were 42 positive *bla*_KPC_ genes, three *bla*_NDM-1_ genes, three *bla*_NDM-5_ genes, one *bla*_NDM-7_ gene, and six *bla*_IMP-4_ genes. Most isolates harbored multiple drug resistance gene, especially as related to extended-spectrum-β-lactamases, *bla*_SHV_, *bla*_TEM_ and *bla*_CTX-M-15_ genes.The resistant gene was transferred into recipient *Escherichia coli* J53 through conjugation in 21.3% (10/47) of the strains. MLST revealed that ST76 (*n* = 36) was the most predominant clone, followed by ST896, ST323 and ST11. A new one ST 2946 was identity by this study.

**Conclusion:**

The carbapenem resistance phenomenon is alarming and *bla*_KPC-2_ is the main resistant gene of CNSE in our hospital. This is the first report of an outbreak caused by *bla*_KPC-2_ positive *K. pneumoniae* ST76 in the Eastern region of Heilongjiang Province, China. Relevant departments should implement infection control and prevention measures to avoid further dissemination of the multi drug-resistant bacteria (MDR).

## Background

The emergence of MDR bacteria is not conducive to infection control. Carbapenems are used as the final treatment agent for infections caused by MDR Gram negative bacteria. Carbapenem resistant *Enterobacteriaceae *(CRE) infection is responsible for a high mortality rate of 26–44% [1]. It has been reported that the high colonization rate of *Klebsiella pneumoniae* Carbapenemase(KPC)-producing *Klebsiella pneumoniae* in patients with ICU is related to the number of comorbidities, administration of carbapenems, β-lactams/lactamase inhibitors, and the time of previous ICU admission [2]. In Asia, the resistance rates of *Enterobacteriaceae* to carbapenems are increasing [3]. The main mechanisms of carbapenem resistance among Enterobacteriaceae are the production of carbapenemases such as KPC and New Delhi metalloβ-lactamase (NDM), extended spectrum β-lactamase (ESBL) or AmpC β-lactamases(AmpC) enzymes that are accompanied more rarely with loss of outer membrane proteins. These resistant genes are often located on plasmids. Timely detection of carbapenem-producing strains is significant for infection control. Here we investigated the prevalence and resistance characteristics of CNSE in the largest university hospital in the eastern region of Heilongjiang Province, focusing on the resistance mechanism and epidemiologic characteristics.

## Methods

### Bacterial strains and Antimicrobial susceptibility testing (AST)

This study was conducted from January 2015 to September 2017. All the *Enterobacteriaceae* isolates were identified and the minimum inhibitory concentrations(MICs) of antibiotics were determined using the Vitek 2 system and the AST-GN card (bioMérieux, France) at the First Affiliated Hospital of Jiamusi University, a 1600 beds hospital. Disk diffusion method was used as a supplementary susceptibility test. As CNSE were considered only those isolates that confirmed as Carbapenems-nonsusceptible (either of ertapenem, imipenem or meropenem) according to Clinical and Laboratory Standard Institutes (CLSI-2016) criteria. *Escherichia coli* ATCC 25922 and *E. coli* J53 (sodium azide resistant) were used as the control for antimicrobial susceptibility test and recipient strain for conjugation experiment, respectively.

### Phenotype experiment

CNSE isolates were evaluated using mCIM as mentioned before [4]. Use a 1 μL inoculation loop to scrape CNSE strain into 2 mL of MH broth and vortex for 15 s. Meropenem disk (10 μg) were immersed in the 35 °C suspension and removed after 4 h of culture. *E. coli* ATCC25922 was formulated into a suspension of 0.5McF bacteria and densely coated on MH plates. The meropenem disk soaked in the bacterial suspension was placed on the MH plate and incubated at 35 °C incubator for 6 h. The results showed that the diameter of the inhibition zone of the meropenem disk soaked in the bacterial suspension was 6-15 mm or there are scattered colonies within the bacterium but with a diameter of 16-18 mm, which is positive for the mCIM test. The MHT was used to screen isolates for the production of carbapenemases, according to CLSI 2016 guidelines.

### DNA extraction

For each CN**S**E, five colonies of each CNSE isolate from overnight culture plates, suspended in 1.5 ml Sodium chloride. The suspension was heated at 100 °C for ten minutes, then centrifuged at 4000 rpm for five minutes to remove cellular debris, and after 100 μl of the supernatant was transferred to a new Eppendorf tube. The DNA was stored at − 20 °C.

### Molecular detection of resistance genes

PCR and nucleotide sequencing techniques were conducted to detect the presence of carbapenemase genes *bla*_KPC_, *bla*_NDM_, *bla*_VIM-1_, *bla*_VIM-2_, *bla*_IMP-4_, *bla*_IMP-8_, *bla*_OXA-48_, *bla*_OXA-23_, *bla*_OXA-24_, *bla*_OXA-51_ and *bla*_OXA-58_, as well as ESBL genes, including *bla*_CTX_, *bla*_TEM_, *bla*_ACC_, and *bla*_SHV_ using primers as described previously [5–7]. Bioedit software was used to analyze test data, and results were compared using online blast software.

### Conjugation experiments

The conjugation experiment was carried out using a membrane bonding experiment as previously described [8]. Both the donor (CNSE) and the recipient strains (*E.coli* J53) were mixed on Luria-Bertani agar at a ratio of 1:3, and the mixtures were incubated for 24 h at 35 °C. Transconjugants were selected in LB broth supplemented with sodium azide (100 μg/ml) and imipenem (1 μg/ml). Colonies which grew on the selective medium were identified by the VITEK-2 Compact system. Strains that harbored carbapenemase and exhibited higher MICs of resistance to carbapenems and cephalosporins than J53 were defined as the transconjugants.

### Homology analysis

MLST was performed using seven housekeeping genes of *K. pneumoniae* which were amplified using primers showed in online databases (http://bigsdb.pasteur.fr/klebsiella/primers_used.html). The products of PCR were sequenced. Sequence types (STs) were determined using online database tools.

### Statistical analysis

The SPSS 22.0 program was conducted using Chi-square test for statistical analysis. *P*<0.05 was statistically significant.

## Results

### Clinical and epidemiological characteristics

**Fig. 1:**
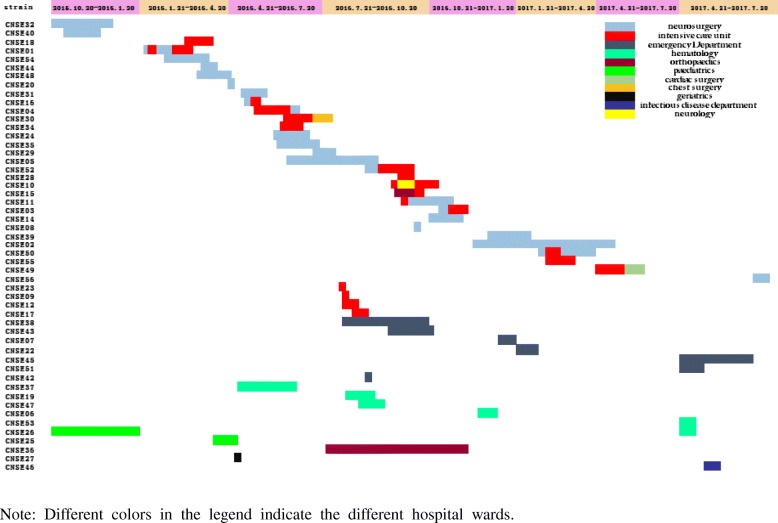
A timeline representing the CNSE isolated in relation to the ward and the duration of stay in each ward

From October 2015 to July 2017 a total of 53 non-duplicated CNSE were isolated from various clinical specimens. The CNSE strains were isolated one from each patient, with age range (5 months - 89 years, median 60.5 years). Among 53 patients 34(64.2%) were male. The mortality of patients with CNSE infections was 22%. Three patients were excluded; one outpatient who had no hospital records and two nosocomial patients who had no longer contact. The majority of the CNSE isolates were *K.pneumoniae* (75.5%, *n* = 40) followed by *Enterobacter cloacae* (9.4%, *n* = 5), *E.coli* (7.5%, *n* = 4), *Klebsiella oxytoca* (3.8%, *n* = 2) whereas, *Citrobacter freundii* and *Serratia marcescens* included one isolate each (1.9%). Specimen with positive culture for CNSE, included sputum (71.7%, *n* = 38), blood (15.1%, *n* = 8), swabs (5.7%, *n* = 3) and urine and pus (3.8%, *n* = 2,each). The CNSE isolates emerged from Neurosurgery 35.8% (*n* = 19), ICU 28.3% (*n* = 15), emergency room 13.2% (*n* = 7), hematology 9.4% (*n* = 5) and other Departments. Department distribution is shown in Fig. 1.

### Antimicrobial susceptibility

According to the VITEK 2 test results, all CNSE isolates were MDR (Table 1). The CNSE isolates were resistant to ampicillin(100%), ampicillin/sulbactam(100%), piperacillin/tazobactam(100%), cefazolin(100%), ceftazidime(100%), ceftriaxone(100%) and cefepime(100%), to amikacin (7.5%), to ciprofloxacin(39.2%) and levofloxacin (24.5%), to aztreonam (96.2%) and to gentamicin and tobramycin (88.7 and 83% respectively).

**Table 1:** Susceptibility to the antimicrobial agents, basic characterization and drug resistance gene of CNSE

Isolate NO.	MIC(ug/mL)														*bla* genotype	MHT/mCIM	strain	Culture type	History of antibiotic use	Invasive procedure	outcome	MLST
	AMP	AMS	TZP	CZO	CAZ	CRO	FEP	ATM	IMP	AMK	GEN	TOB	CIP	LEV								
CNSE01 CNSE02 CNSE15	R	R	R	R	R	R	R	R	R	S	R	R	R	S	KPC-2, SHV, TEM, CTX-M-15	+/+	KP	Sputum	YES	YES	Survived	ST76
CNSE03 CNSE09 CNSE12 CNSE44 CNSE48	R	R	R	R	R	R	R	R	R	S	R	R	I	S	KPC-2, SHV, TEM, CTX-M-15	+/+	KP	Sputum	YES	YES	Died	ST76
CNSE04 CNSE05 CNSE07 CNSE14 CNSE16 CNSE29 CNSE30 CNSE31 CNSE32 CNSE34 CNSE35 CNSE54	R	R	R	R	R	R	R	R	R	S	R	R	I	S	KPC-2, SHV, TEM, CTX-M-15	+/+	KP	Sputum	YES	YES	Survived	ST76
CNSE06	R	R	R	R	R	R	R	R	R	S	S	S	S	S	KPC-2, SHV	+/+	KP	Sputum	YES	YES	Died	ST323
CNSE08 CNSE11	R	R	R	R	R	R	R	R	R	S	R	R	R	R	KPC-2, SHV, TEM, CTX-M-15	+/+	KP	Sputum	YES	YES	Survived	ST76
CNSE10	R	R	R	R	R	R	R	R	R	S	R	R	I	S	KPC-2, SHV, TEM, CTX-M-15	+/+	KP	Sputum	NO	NO	Survived	ST76
CNSE17	R	R	R	R	R	R	R	R	R	S	R	R	S	S	KPC-2, SHV, CTX-M-15	+/−	KP	Sputum	YES	YES	Died	ST76
CNSE18	R	R	R	R	R	R	R	R	R	S	R	R	I	S	KPC-2, SHV, CTX-M-15	+/+	KP	Sputum	YES	YES	Died	ST76
CNSE19	R	R	R	R	R	R	R	R	R	S	S	I	R	R	KPC-2, NDM-5, CTX-M-14, TEM	−/+	EC	Blood	YES	YES	Survived	ND
CNSE20	R	R	R	R	R	R	R	R	R	S	R	R	I	S	KPC-2, NDM-5, SHV, CTX-M-177, TEM	+/+	KP	Sputum	YES	YES	Died	ST76
CNSE22	R	R	R	R	R	R	R	R	R	S	R	I	S	S	IMP-4, SHV, TEM	+/+	KP	Sputum	YES	YES	Died	ST896
CNSE23	R	R	R	R	R	R	R	R	R	S	R	R	R	R	KPC-2, SHV, TEM, CTX-M-15	+/+	KP	Blood	YES	YES	NR	ST76
CNSE24	R	R	R	R	R	R	R	R	R	S	R	R	I	S	KPC-2, SHV, TEM, CTX-M-15	+/+	KP	Blood	YES	YES	Survived	ST76
CNSE25	R	R	R	R	R	R	R	R	R	S	R	R	R	R	NDM-1, TEM, CTX-M-15	+/+	EC	Sputum	YES	YES	Survived	ND
CNSE26	R	R	R	R	R	R	R	S	R	S	S	S	S	S	IMP-4	+/+	KO	Sputum	YES	NO	Survived	ND
CNSE27	R	R	R	R	R	R	R	R	R	R	R	R	R	R	NDM-7, TEM, CTX-M-15	−/+	EC	Urine	YES	YES	Survived	ND
CNSE28	R	R	R	R	R	R	R	R	R	S	R	R	I	S	KPC-2, SHV, TEM, CTX-M-15	+/+	KP	Blood	YES	YES	Died	ST76
CNSE36	R	R	R	R	R	R	R	R	R	S	R	R	R	I	KPC-2, SHV, TEM, CTX-M-15	+/+	KP	Secretion	YES	YES	Survived	ST76
CNSE37	R	R	R	R	R	R	R	R	I	S	R	I	S	S	IMP-4, SHV, TEM, CTX-M-15	+/+	KP	Sputum	YES	YES	Died	ST2964
CNSE38	R	R	R	R	R	R	R	R	R	S	R	R	S	S	NDM-1, SHV, CTX-M-15	−/+	ECL	Secretion	YES	YES	Survived	ND
CNSE39	R	R	R	R	R	R	R	R	R	S	S	S	S	S	KPC-2, SHV, TEM, CTX-M-15	+/+	KP	Sputum	YES	YES	Survived	ST76
CNSE40	R	R	R	R	R	R	R	R	R	S	R	R	I	S	KPC-2, SHV, TEM, CTX-M-15	+/+	KO	Sputum	YES	YES	Survived	ND
CNSE41	R	R	R	R	R	R	R	R	S	S	R	R	R	S	–	−/+	SM	Secretion	NR	NR	NR	ND
CNSE42	R	R	R	R	R	R	R	R	I	R	R	R	S	S	IMP-4, TEM	+/+	ECL	Blood	YES	YES	Died	ND
CNSE43	R	R	R	R	R	R	R	R	R	S	R	R	R	R	KPC-2, SHV, TEM, DHA	+/+	ECL	Sputum	YES	YES	Died	ND
CNSE45	R	R	R	R	R	R	R	R	R	R	R	R	R	R	KPC-2, TEM	+/+	CF	pus	YES	YES	Survived	ND
CNSE46	R	R	R	R	R	R	R	R	R	S	R	I	R	R	NDM-1 IMP-4, SHV, TEM, CTX-M-15	+/+	ECL	Urine	YES	YES	Survived	ND
CNSE47	R	R	R	R	R	R	R	R	R	S	S	S	R	R	KPC-2, SHV	−/+	KP	Blood	YES	YES	Survived	ST11
CNSE49	R	R	R	R	R	R	R	R	R	S	R	R	R	S	KPC-2, SHV, TEM, CTX-M-15	+/+	KP	Blood	YES	YES	Survived	ST76
CNSE50	R	R	R	R	R	R	R	R	R	S	R	R	R	R	KPC-2, TEM, CTX-M-15	+/+	KP	Sputum	YES	YES	Survived	ST76
CNSE51	R	R	R	R	R	R	R	R	R	S	S	S	R	R	KPC-2, IMP-4, TEM, DHA	+/+	ECL	pus	YES	YES	Survived	ND
CNSE52	R	R	R	R	R	R	R	R	R	S	R	R	I	S	KPC-2, TEM, CTX-M-15	+/+	KP	Sputum	YES	YES	Survived	ST76
CNSE53	R	R	R	R	R	R	R	S	R	R	R	R	R	R	NDM-5, SHV	+/+	EC	Blood	YES	YES	Died	ND
CNSE55	R	R	R	R	R	R	R	R	R	S	R	R	R	S	KPC-2, SHV, TEM, CTX-M-15	+/+	KP	Sputum	YES	YES	NR	ST76
CNSE56	R	R	R	R	R	R	R	R	R	S	R	R	R	S	SHV, TEM, CTX-M-15	+/+	KP	Sputum	YES	YES	Survived	ST76

*AMP* ampicillin, *AMS* ampicillin-sulbactam, *TZP* piperacillin/tazobactam, *CZO* cefazolin, *CAZ* ceftazidime, *CRO* ceftriaxone, *FEP* cefepime, *ATM* aztreonam, *IMP* imipenem, *GEN* gentamicin, *TOB* tobramycin, *AMK* amikacin, *CIP* ciprofloxacin and *LEV* levofloxacin, *KP Klebsiella pneumoniae, EC Enterobacter cloacae, EC Escherichia coli*, *KO Klebsiella oxytoca*, CF *Citrobacter freundii*, *SM Serratia marcescens*, *NR* No Record, *ND* Not Determined

### Phenotypic experiment

According to mCIM results, the positive strains were 52 (98.1%). The positive rate of MHT was 90.6%, also shown on Table 1. There was no statistically significant difference between mCIM and MHT in their ability to detect carbapenemase (*p* = 0.219).

### Drug resistant genes

The results from the study of *bla*genes among CNSE isolates were listed on Table 1. Among 53 CNSE isolates, 42(79. 2%) were KPC-2 producers. Six isolates (11.3%) including three *E. cloacae*, two *K. pneumoniae* and one *K.oxytoca* carried *bla*_IMP-4_ genes. Seven isolates (13.2%) produced NDM carbapenemase. One *E.coli* and two *E.cloacae* carrying *bla*_NDM-1_, two *E.coli*, one *K.pneumoniae* carrying *bla*_NDM-5_, one *E.coli* carrying *bla*_NDM-7_. ESBL genes were found in 43(81.1%), 43 (81.1%) and 45 (84.9%) isolates carried *bla*_SHV_, *bla*_CTX-M_ and *bIa*_TEM_, respectively. Thirty-three *K. pneumoniae* carried *bla*_SHV_, *bla*_CTX-M_ and *bla*_TEM_ genes simultaneously. Other drug resistance genes (*bla*_VIM_, *bla*_OXA-58_, *bla*_OXA-51_, *bla*_OXA-48_, *bla*_OXA-23_, *bla*_OXA-24_ and *bla*_ACC_) were not detected.

### Horizontal transfer of *bla*_KPC_ and *bla*_NDM_

Forty seven *bla*_NDM_ positive or *bla*_KPC_ positive isolates were selected for conjugation. The conjugation experiments showed that the plasmids with *bla*_NDM_ and *bla*_KPC_ from ten CNSE isolates were successfully transferred to recipient *E. coli* J53. The success rate was 21.3%, and the conjugants exhibited higher resistance to carbapenems compared to J53. The MICs of imipenem and ertapenem for the conjugants ranged from 0.12 to 16 mg/L (Table 2).

**Table 2:** The MICs of Cephalosporins and carbapenems for the conjugants

Isolate NO.	MIC					Drug-resistant gene transfer
	CAZ	CRO	FEP	IMP	ETP	
J53	≤0.12	≤0.25	≤0.12	≤0.25	≤0.12	–
J_CNSE09_	4	32	0.5	≤0.25	0.5	KPC
J_CNSE15_	16	32	4	0.5	0.5	KPC
J_CNSE18_	≤0.12	1	≤0.12	0.5	≤0.12	KPC
J_CNSE19_	≥64	≥64	8	8	≥8	NDM
J_CNSE24_	0.25	≤0.25	≤0.12	≤0.25	≤0.12	KPC
J_CNSE27_	≥64	≥64	16	≥16	≥8	NDM
J_CNSE28_	16	16	≤0.12	0.5	≤0.12	KPC
J_CNSE35_	16	32	16	≤0.25	≤0.12	KPC
J_CNSE38_	≥64	≥64	16	≥16	≥8	NDM
J_CNSE40_	4	32	≤0.12	≤0.25	≤0.12	KPC

### Homology analysis

**Fig. 2:**
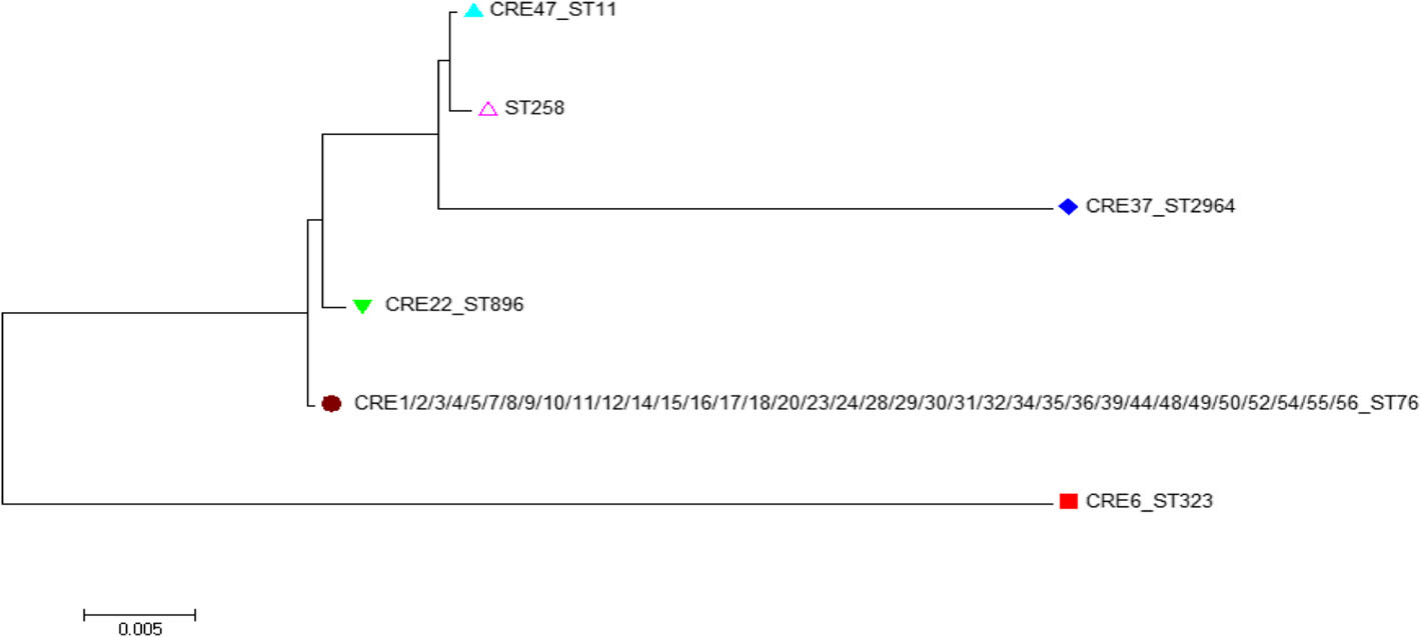
Phylogenetic tree of 40 *Klebsiella pneumoniae* isolates

All of five distinct MLST sequence types were observed among the forty CRKP isolates, including ST76 (*n* = 36), ST11 (*n* = 1), ST323 (*n* = 1), ST896 (*n* = 1) and ST2964 (*n* = 1). CRKP ST76 isolates were isolated, mainly from neurosurgery (50.0%) and intensive care unit (41.7%). The ST2964 isolate was a novel type found in this study and its DNA sequences were submitted into the Institute Pasteur MLST database for *K. pneumoniae* (available at http://bigsdb.pasteur.fr/klebsiella/klebsiella.html). ST76 has earlier evolution than ST896, ST2964 and ST11. Their evolutionary relationships are shown in Fig. 2.

## Discussion

The increase in ESBL-producing bacteria indicates a rise in the use of carbapenems and this may be the reason for the increasing number of carbapenem resistant enterobacteriaceae in recent years [9]. As the mortality in present study was found as high as 22% (11/50), it was necessary to evaluate the carbapenem resistance trend in our hospital and the gene characteristics responsible for the resistance. Clinical treatment using carbapenem may vary depending on the mechanism of resistance [10].

As it is well known, the production of KPC-type enzyme is the most important mechanism of carbapenem resistance in Enterobacteriaceae. KPC-producing *Enterobacteriaceae spp.* have emerged globally. *Bla*_KPC_ is now endemic all over the world. In Europe, especially Austria, Germany, Greece, Russia and United Kingdom; In Latin America, including Brazil and Mexico;The Asia-Pacific, China, Japan, Taiwan; Also found in Israel and the United States [11]. It has disseminated dramatically throughout China, causing serious infectious diseases. In China, the first case of *bla*_KPC_ was found in Hangzhou, which was isolated from one elderly patient’s sputum in 2004 [12]. Currently, *bla*_KPC_ has been reported in many regions of China, including Beijing, Zhejiang, Taiwan, and Sichuan [13–16]. To the best of our knowledge, this article is the first report on the prevalence of CNSE in the eastern part of Heilongjiang province.

Metallo-β-lactams, including IMP and NDM, contributed to carbapenem resistance in Enterobacteriaceae, particularly NDM-1, which has caused a global health threat. NDM isolates are highly resistant to many classes of antibiotics and have the potential to spread rapidly to many members of Enterobacteriaceae which can cause nosocomial outbreaks [17]. *Bla*_NDM-1_ was first reported in *K.pneumoniae* from a Swedish patient in 2009 [18]. It exists in many variant forms and has been reported in Africa, Europe, Australia, America and Asia, specifically Turkey, Algeria, France, Italy, Greece, New Zealand, Mexico and China [18–26]. Chen et al. first reported the emergence of *bla*_NDM-1_-positive strains in four different provinces in China [27] . *Bla*_NDM-1_ widely disseminated in China, the epidemic dissemination of NDM-1-producing *Enterobacteriaceae spp.* has been confirmed in Henan, Zhejiang, Yunnan, Hunan and other provinces [28–31]. One major reason for this is the rapid dissemination of the *bla*_NDM-1_ gene is plasmid location [29]. In our study four *E. coli*, two *E. cloacae* and one *K. pneumoniae* were the seven isolates carrying *bla*_NDM_.

In present study CNSE have been found to carry at least two different types of beta-lactamase resistance genes, most isolates (58.5%) carried simultaneously the *bla*_KPC-2_, *bla*_CTX-M-15_, *bla*_SHV_, *bla*_TEM_ gene, this is consistent with Netikul’s report [32]. The strains that produce KPC can caused the mediation of carbapenem resistance in *K. pneumoniae* [33]. Most of the strains were resistant to a variety of antimicrobial agents, including cephalosporins, carbapenems, enzyme inhibitors and so on. Indicates that the phenomenon of drug resistance was serious.This may be related to clinicians’ experience of medication. CTX-M can hydrolyze cephalosporin and mono-amide antibiotics, CTX-M exhibits powerful activity against ceftriaxone [34]. According to amino acid sequence similarities, *bla*_CTX-M-15_ is a member of the *bla*_CTX-M-1_ subgroup. The hydrolysis of ceftazidime by CTX-M-15 was strongest. As is well known, TEM can hydrolyze penicillin and first generation cephalosporin. SHV is resistant to cephalosporin and aztreonam, in particular, it shows drug resistance to third-generation cephalosporins, and some were resistant to beta-lactamase inhibitors. The antimicrobial resistance may be produced by a variety of beta-lactamase genes which enhances antimicrobial resistance and broadens the resistant spectrum and may be the reason why drug resistance of these strains was very strong [35]. Horizontal genes transfer tend to be associated with the high prevalence of ESBLs and the increasing presence of carbapenemases [36]. Conjugation was the reason for carbapenem resistance acquisition in carbapenem resistant enterobacteriaceae [37]. Acquired carbapenemases are encoded by genes located in mobile elements such as transposons and plasmids, which may transfered to different strains and species [38]. Interestingly, not all carbapenemase-producing isolates are carbapenem-resistant [39], this is because, while production of carbapenemase always elevates the MICs of carbapenems, they may not be high enough to be classified as resistance or intermediate resistance [40].

The MHT and mCIM results for phenotypic testing were recommended by CLSI for carbapenemase detection and early screening of carbapenemases to avoid the further spread of resistant bacteria. We evaluated the effectiveness of mCIM and our results indicated that mCIM had high sensitivity and specificity for detecting carbapenemase-producing Enterobacteriaceae. Although one strain gave false positive result in mCIM. False positive results may be due to different resistance mechanisms in carbapenem resistance. Our results showed that MHT had powerful ability to detect carbapenemase positive isolates 48 (90.6%). Among them only four isolates were *bla*_NDM_ positive and six others were *bla*_IMP_. Many studies have showed that MHT often has low sensitivity and specificity for detection of class B carbapenemases [41], and this is consistent with our results. There was no statistical significance in the detection of carbapenems between MHT and mCIM. They are all the particularly useful tool for the detection of carbapenemase producers.

Isolates with the *bla*_KPC_ gene are spread in many regions such as North America, Latin America and Asia with ST11 and ST258 being the common types [42–44]. The epidemiological information on CRKP in the eastern region of Heilongjiang Province is unknown. Interestingly, *bla*_KPC_ gene has been detected in our CRKP isolates but none of the isolates were ST11 which were reported in other parts of China. In our study, we demonstrated that the sequence type ST76 was the predominant type among CRKP isolates which are *bla*_KPC-2_ positive in Heilongjiang Province. To the best of our knowledge, the ST76 clone has been sporadically reported in Taiwan and America [45, 46]. In Japan, ST76 has been isolated from meat [47]. Zhu reported in 2014 an outbreak caused by NDM-1-producing *K. pneumoniae* ST76 in neonates in Shanghai [48]. Figure 1 shows the outbreak of CNSE in the department of neurosurgery and ICU of our hospital exhibiting sporadic phenomena in other departments. During the study period, the first case of type ST76 CRKP was detected from a sample coming from the department of neurosurgery.

In addition, several other ST types were found among CRKP isolates, including ST323, ST11, ST896 and the novel ST2964 clone. There are not many reports about ST323 and ST896. ST76 type evolution before ST2964. The common ST258 in the United States differs from the common ST11 in China only by a tonB housekeeping gene, with only a difference of four bases, so the two are closely related. MLST studies indicated that clones of CRKP were widespread. Here, we emphasize the need to actively monitor the spread of CRKP. Our findings supplied the novel epidemiologic data of CRKP in China.

At the beginning of the epidemic, most of the strains were isolated from respiratory tract specimens, which suggested that respiratory tract colonization had occurred. Almost all the patients had one or more severe conditions, of which 19 (35.8%) patients suffered from sudden cerebral hemorrhages, six (11.3%) patients severe head injury caused by trauma, 11 (20.7%) patients patients with myelodysplastic syndrome and others with cancer of the stomach, abdominal closed injury, acute cerebral infraction among other diseases. In our study, 50 (94.3%) patients were treated with invasive procedures, such as the arteriovenous catheter, tracheotomy, ventilator-assisted breathing, among other procedures. Fifty-one patients (96.2%) had a history of antibiotic exposure, which contributed to the production of CNSE. In addition, receiving a transplant was an independent factor in CNSE infection [49]. The prevention and control of CNSE infection or colonization mainly includes the following aspects: necessary interventions for the hand hygiene of medical workers and patients; routine ongoing active monitoring;prescribing medications strictly in accordance with the regulations for the rational use of antimicrobial agents; and isolated infection or colonization of CNSE patients. Currently, a collaboration is needed between the pharmacists, microbiologists, infection control practitioners, and infectious disease clinicians in working together to defeat CNSE [50].

As it is well known, the economy is underdeveloped in the eastern part of Heilongjiang Province and the prevalence of CNSE in such area must be treated with the needed urgency. Therefore, there is an urgent need to establish more and better surveillance in our hospital to prevent further spread of the resistant bacteria.

## Conclusions

We report for the first time, the endemic spread of CNSE in the largest university hospital in the eastern region of Heilongjiang Province, China. Even if the prevalence of CNSE were low, we should still be monitored closely. The main drug resistance genes are *bla*_KPC-2_, *bla*_NDM_, *bla*_TEM_, *bla*_SHV_ and *bla*_CTX-M-15_. The spread of CRKP was caused by the selection of *bla*_KPC-2_ positive *K. pneumoniae* ST76 epidemic clones. This will help prevent the widespread spread of CNSE in this hospital and provide a basis for the epidemiology of CNSE in Heilongjiang Province. More research should be done to better understand the causes of CNSE.

## Abbreviations

AmpC: AmpC β-lactamases
CLSI: Clinical and Laboratory Standard Institutes
CNSE: Carbapenem non-susceptible Enterobacteriaceae
CRKP: Carbapenem-resistant *Klebsiella pneumoniae*
ESBL: Extended spectrum β-lactamase
KPC: *Klebsiella pneumoniae* Carbapenemase
mCIM: Modified carbapenem inactivation method
MHT: Modified hodge test
MICs: Minimum inhibitory concentrations
MLST: Multilocus sequence typing
NDM-1: New Delhi metalloβ-lactamase-1
PCR: Polymerase chain reaction
STs: Sequence types
